# Ablation combined with video-assisted thoracic surgery hybrid technique for multiple primary lung cancer

**DOI:** 10.1016/j.isci.2025.112703

**Published:** 2025-05-20

**Authors:** Ruifeng Xu, Guochao Zhang, Yun Che, Na Ren, Shuaibo Wang, Chenglin Yang, Qi Xue, Fengwei Tan, Liang Zhao, Jie He

**Affiliations:** 1 Department of Thoracic Surgery, National Cancer Center/National Clinical Research Center for Cancer/Cancer Hospital, Chinese Academy of Medical Sciences and Peking Union Medical College, Beijing 100021, China; 2 Department of Thoracic Surgery, Beijing Institute of Respiratory Medicine and Beijing Chao-Yang Hospital, Capital Medical University, Beijing 100020, China; 3 National Cancer Center/National Clinical Research Center for Cancer/Cancer Hospital and Shenzhen Hospital, Chinese Academy of Medical Sciences and Peking Union Medical College, Shenzhen 518116, China

**Keywords:** Surgery, Medical procedure, Cancer

## Abstract

Increased public health awareness and expanded low-dose computed tomography (CT) utilization, accelerated by the COVID-19 pandemic, have elevated detection rates of pulmonary ground-glass nodules (GGNs). Patients with multiple primary lung cancer (MPLC) often present with multiple GGNs, posing challenges for precise treatment and prognostic assessment. Current therapies including stereotactic body radiation therapy (SBRT), chemotherapy, and immunotherapy face efficacy and safety limitations. While video-assisted thoracic surgery (VATS) is the primary treatment for high-risk GGNs, the sole reliance on surgery may cause excessive loss of lung function. Image-guided thermal ablation techniques can effectively treat smaller lesions with lung preservation. This review explores molecular mechanisms of ablation, VATS-ablation synergy, and the potential value of this approach in combination with immunotherapy. The clinical application prospects, including advancements in navigation techniques and equipment, are also discussed. Overall, this hybrid surgical strategy represents a promising option for patients with multiple lesions, minimizing lung function loss and the psychological burden.

## Graphical abstract

**Figure:**
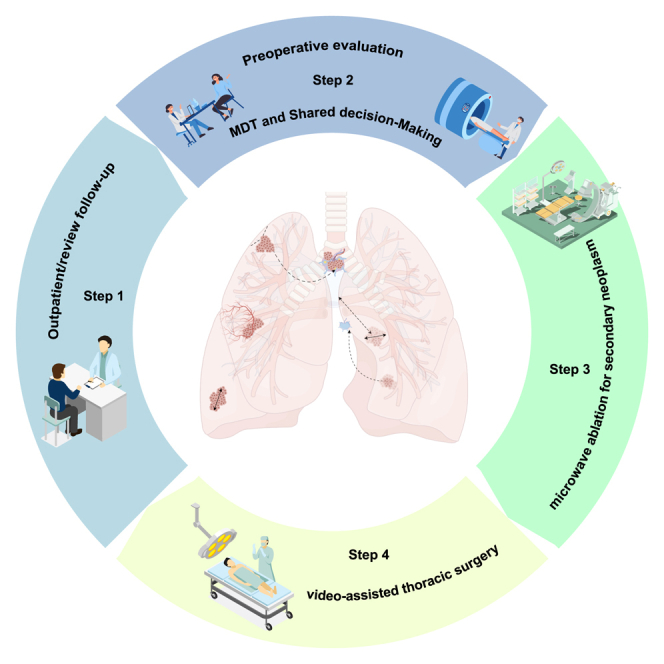

## Introduction

Currently, lung cancer still has the highest incidence among malignant tumors in the world and is the leading cause of tumor-related deaths.^1^ As public health awareness has increased in recent years and with the spread of COVID-19, computed tomography (CT) scanning has been widely used in the population, leading to a gradual increase in the rate of detection of primary lung cancer characterized by ground-glass nodules (GGNs), especially multiple primary lung cancer (MPLC), which is characterized by multiple GGNs.

MPLC refers to the simultaneous or sequential occurrence of two or more primary malignant tumors in the same patient’s lungs. MPLC can also be classified into synchronous multiple primary lung cancer (sMPLC) and heterochronous multiple primary lung cancer (hMPLC) according to the time interval between the occurrence of different foci, histological characteristics, and other characteristics. sMPLC accounts for approximately 2.6%–7.9% of cases in patients undergoing surgery for non-small cell lung cancer.^2^^,^^3^^,^^4^^,^^5^^,^^6^ According to the Martini-Melamed (M-M) criteria,^7^ sMPLC refers to (1) Multiple isolated tumors occurring at the same time, which may be of the same or different pathological types. (2) In cases of identical pathology, the tumors are generally distributed across different lung segments, lobes, or sides of the lung, and the individual tumors are of an *in situ* origin and without evidence of extrapulmonary metastasis or lymphatic spread.^8^ In clinical practice, an important imaging characteristic of MPLC is the presence of multiple GGNs.

According to relevant statistics, approximately 50% of GGN patients have multiple GGNs.^9^^,^^10^^,^^11^ Although the academic community regards lung cancers with GGN characteristics as a less active subtype of lung adenocarcinoma,^12^ 13% of cases of GGN progression are still detected within a 10-year follow-up.^13^ In contrast, in postsurgical patients with MPLC featuring multiple GGNs, the presence of one residual GGN is followed by the subsequent growth of other residual GGNs in 41% of patients.^14^ The ability to effectively manage these residual GGNs is critical to the prognosis of MPLC patients.^15^^,^^16^^,^^17^ The aforementioned patients with multiple MPLC characterized by multiple GGNs are different from patients with single nodules in terms of clinicopathological features and treatment,^18^ but their precise treatment is also one of the difficulties in the current field of lung cancer research.

**Figure 1:**
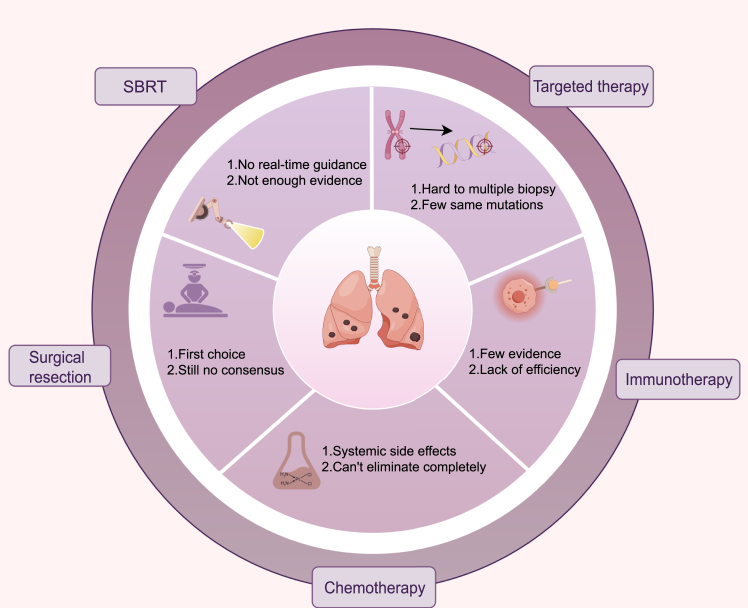
Dilemma regarding the current major treatments for MPLC

Although many types of treatments are available for patients with MPLC, these strategies have limitations in clinical practice (Figure 1). To date, insufficient evidence has been reported for the effectiveness of stereotactic body radiation therapy (SBRT) in the treatment and prediction of the prognosis of patients with MPLC.^19^^,^^20^ Nevertheless, radiological dose delivery and real-time image guidance are difficult to perform simultaneously. Moreover, chemotherapy can scarcely eliminate all cancer cells, and the systemic side effects should not be ignored. Targeted therapy plays a significant role in the comprehensive treatment of lung cancer, but obtaining pathological results for multiple lesions from patients with MPLC is still difficult.^21^^,^^22^ The pathological results for the multiple lesions may not reveal the same mutation even when the samples are obtained via percutaneous biopsy. Furthermore, more evidence is still needed to demonstrate the effectiveness in immunotherapy (PD-1/PD-L1) against MPLC.^23^

Currently, video-assisted thoracic surgery (VATS) remains the main option for the treatment of high-risk GGNs diagnosed via non-invasive techniques, such as imaging^24^; Nonetheless, if surgery is used as the only option for the management of all GGNs, an unnecessary loss of lung function may occur. How to eliminate satellite lesions effectively from MPLC and reduce the psychological burden on patients while preserving lung function as much as possible is an essential issue in clinical practice.^25^^,^^26^^,^^27^ Several studies have confirmed that image-guided thermal ablation (IGTA) (including radiofrequency ablation [RFA], microwave ablation [MWA], and laser ablation) and cryoablation are effective methods for treating GGNs with smaller diameters,^28^^,^^29^^,^^30^^,^^31^^,^^32^^,^^33^^,^^34^^,^^35^^,^^36^ as thermal ablation (e.g., RFA, MWA, and laser ablation) induces cell death via hyperthermia, whereas cryoablation uses hypothermia. However, the application value of VATS combined with hybrid ablation surgery for MPLC and the relevant medical-industrial integration value still need to be further investigated.

In this review, we introduce tumor ablation techniques and their associated molecular mechanisms. In addition, we discuss the background of the emergence, value of the combination, and prospects for the development of ablation techniques combined with VATS hybrid surgery.

## Background on the emergence of VATS combined with thermal ablation surgery

### VATS alone has limitations in current applications

In clinical practice, surgically resected lesions predominantly are tumor lesions with the following characteristics: more solid components, larger size, vascular or tracheal penetration, abnormal lymph node enlargement in the drainage area, pleural traction, etc., and significant signs of malignancy.

Surgical management has undergone remarkable progress in the twentieth century. The gold standard of extent resection has evolved from pneumonectomy to lobectomy or even sublobar resection. For patients with GGNs, wedge resection is generally preferred. Nevertheless, the incisional approach has evolved from rib-spreading thoracotomy to 3-port VATS, uniportal VATS, or even robotic-assisted thoracic surgery. Surgical treatments are performed in a minimally invasive fashion.^37^

**Figure 2:**
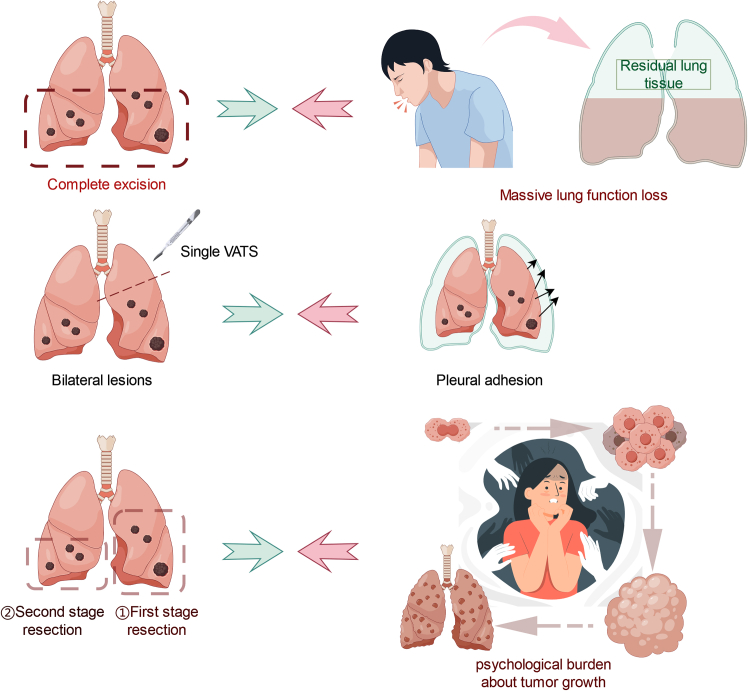
Limitations of using thoracoscopy alone to manage multiple primary lung nodules

However, for patients with multiple GGNs, premature surgical resection with VATS is associated with a series of contradictions (Figure 2).

(1) First, there is a contradiction between the extensive early resection of multiple GGNs and the preservation of postoperative lung function. The pathological types of GGNs are divided into alveolar epithelial atypical adenomatous hyperplasia (AAH), adenocarcinoma *in situ* (AIS), microinvasive lung adenocarcinoma (MIA), and invasive adenocarcinoma (IAC).^38^^,^^39^^,^^40^^,^^41^^,^^42^ For preinvasive lesions (lesions prior to IAC) in patients with MPLC, extensive early surgical resection does not significantly improve overall survival compared with elective surgery after 3–4 months of follow up. However, the premature surgical resection of a large number of nodules can cause early organ damage, a loss of lung function, a decrease in a patient’s postoperative quality of life, and a risk of perioperative complications.^18^.
(2) Second, there is a contradiction between bilateral multiple primary lesions and the unilateral extent to which the VATS procedure can be performed at one time. For patients with multiple bilateral MPLC nodules, thoracoscopic surgery cannot be used to resect multiple bilateral lung nodules within a short period. In addition, the number of surgical resections is limited because extensive and significant pleural adhesions can occur after surgery.
(3) Finally, the contradiction between staged surgery for multiple bilateral nodules and the psychological burden caused by lesion progression must be addressed. Elderly patients or patients with underlying diseases have a poorer baseline function and consequently cannot tolerate the simultaneous resection of multiple MPLC nodules. However, resection of only some GGN lesions is associated with a greater psychological burden due to fear among tumor patients, and second-stage surgery is more difficult.^25^^,^^26^^,^^27^ Therefore, for MPLCs with different pathological types, distant anatomical locations, and many nodules, a combination of therapeutic strategies is needed in practice.

### Thermal ablation and VATS have good concurrent indications and efficacy in MPLCs

Currently, the main nonsurgical treatment modalities for MPLC include chemotherapy, immunotherapy, SBRT, and ablation therapy (Table 1). Among them, chemotherapy and immunotherapy are important forms of adjuvant and neoadjuvant therapy and are mainly applied to control residual tumor cells after surgery or to downstage lesions before surgery. However, chemotherapy and immunotherapy can induce systemic side effects and cannot radically eliminate tumor cells^43^; thus, these treatments are not suitable for combination with surgery. In contrast, SBRT has only local side effects and can control local lesions to a certain extent; however, SBRT equipment requires specific radiation shielding, which conventional surgical platforms find difficult to accommodate. Therefore, SBRT is not suitable for combination with thoracoscopic surgery. Ablation therapy can locally eradicate lesions, and conventional bedside CT or electromagnetic navigation can meet the localization requirements. Additionally, VATS can be used to observe the lesion treatment status after ablation therapy and simultaneously assist in managing short-term complications of ablation therapy. Therefore, when combined with VATS, ablation therapy has good applicability in the management of MPLC. Notably, this article primarily focuses on preoperative CT/ENB-guided ablation (targeting lesions inaccessible during VATS) rather than intraoperative ablation (performed under direct visualization via VATS), as the latter may allow for complete resection of lesions during the surgical procedure itself. This distinction emphasizes the complementary role of preoperative ablation in managing lesions that cannot be addressed through standard VATS resection, thereby preserving lung function while ensuring oncological efficacy.

**Table 1:** Comparison of nonsurgical treatments for lung cancer

Nonsurgical treatment	Capacity for eradication	Side effects	Combination with surgical treatment
Chemotherapy	No	Systemic side effects	Pre- or postoperative, or intrapleural perfusion hyperthermia
Immunotherapy	No	Systemic side effects	Pre- or postoperative
**CT/ENB-guided ablation therapy**	**Yes**	**Local sideeffects**	**Can be performed concurrently with surgery (pre- or postoperative ablation)**
Stereotactic body directed radiotherapy (SBRT)	Yes	Local side effects	Independent of surgery
Target therapy	No	Systemic side effects	Pre- or postoperative, need for pathology gene detection.

Ablative therapy has shown noninferiority in terms of the prognosis of stage I non-small cell lung cancer (NSCLC). In a meta-analysis including 792 patients, it was shown that there was no significant difference between ablative therapy and surgery in terms of overall survival (OS) and tumor-specific survival (CSS) from 1 to 5 years, or disease-free survival from 3 to 5 years.^44^^,^^45^^,^^46^^,^^47^^,^^48^^,^^49^^,^^50^^,^^51^ The advantages of combining ablative therapy include its minimal to no effect on lung function compared with that of conventional surgical treatment and its lower costs associated with both single treatment and cumulative treatment for stage I NSCLC over a period of 1 year.^52^

## Introduction to the principles and molecular mechanisms of thermal ablation and cryoablation

Unlike surgical resection, which cuts into the tissue surrounding the tumor, direct destruction of the tumor tissue by ablation causes a more significant immune response.^53^^,^^54^^,^^55^^,^^56^^,^^57^^,^^58^^,^^59^^,^^60^^,^^61^^,^^62^ Hence, hybrid surgery may provide a better postoperative antitumor effect. Knowing the molecular mechanism of ablation may help in understanding the principles of hybrid surgery.

### Mechanism of ablative techniques

Ablation is a type of energy-dependent therapy, the main mechanism of which is the precise and minimally invasive induction of irreversible damage or coagulative necrosis of tumor cells in focal tissues through the biological effects of hyperthermia (such as RFA, MWA, and laser ablation) or hypothermia (such as cryoablation), which subsequently causes local inflammatory responses.^63^^,^^64^

Currently, the thermal ablation techniques applied to lung tumors include RFA, MWA, cryoablation, and laser ablation. RFA, MWA, and cryoablation are the main techniques used. Table 2 compares the detailed differences among these three ablation techniques.

**Table 2:** Comparison of the advantages, disadvantages and molecular mechanisms of ablation techniques

Technology type	Principle	Advantages	Disadvantages	Molecular mechanisms in the central region	Molecular mechanisms of transitional regions
RFA	Radio frequency alternating current (60°C–100°C)	1. Electrode conformity is good. 2. Can protect the peripheral organs. 3. Early development, currently widely used	1. Highly affected by thermal convection	1. Hyperthermia-induced changes in cell membrane fluidity and permeability; impaired substance transport. 2. Hyperthermia-induced changes in mitochondrial morphology and function, and impaired energy metabolism.	1. Leakage of damage-associated molecular patterns (DAMPs; DNA, RNA, HSPs, etc.) triggers programmed cell death and the activation of the intrinsic immune response. 2. Cytokine release and activation of adhesion factors caused by the destruction of the extracellular matrix around the tumor.
MWA	Microwave electromagnetic field (60°C–150°C)	1. High energy, large range, rapid ablation. 2. Small thermal deposition effect, suitable for large perivascular ablation.	1. The application was developed late and needs promotion.		
Laser Ablation	Photonic-to-thermal energy conversion (the temperature cannot be determined)	1. Precisely directed via optical fiber catheters, enabling controlled ablation boundaries.^65^ 2. Minimal tissue carbonization occurs during photonic-to-thermal energy conversion.	1. Limited ablation range: the single ablation volume is relatively small (8–10 mm).^66^ 2. Lower efficiency: a prolonged treatment duration and susceptibility to tissue heterogeneity.		
Cryoablation	Heat absorption by gasification (−140°C to 20°C–40°C, argon–helium, or −196°C–80°C liquid nitrogen)	1. Rarely causes local pain, suitable for patients with pleural and periosteal tumors. 2. The boundaries of the ice sphere are clear.	1. Depletes the patient’s platelets and is not suitable for patients with poor coagulation function.	1. Freezing of extracellular water induced transient extremely low temperatures; the formation of a local osmotic gradient and a solution effect leading to cell membrane disruption. 2. Local microvascular constriction around the tumor, causing ischemic cell necrosis.	1. Cellular autophagy triggers the exposure of tumor-associated antigens, further leading to specific antitumor immunity.

**Figure 3:**
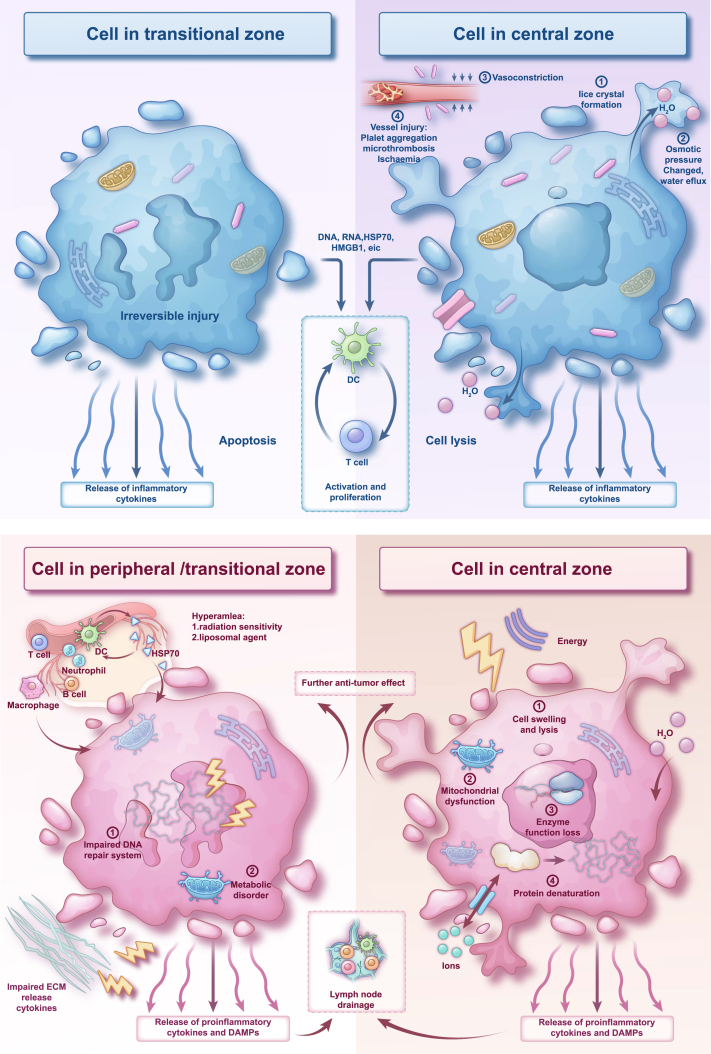
Mechanisms of thermal ablation and cryoablation

The general effect of thermal ablation or cryoablation on tumor cells can be divided into three regions^67^: the central region, the migrating region, and the surrounding unaffected tissue (Figure 3). The central region is the area around the ablation needle where coagulative necrosis directly occurs; the migrating region shows apoptosis or reversible cellular damage, mainly due to heat transfer from the central region; and the peripheral tissues are those in the periphery of the migrating zone that are not affected by the energy.

While the figure depicts shared mechanisms, the specific temperature ranges and immune effects vary by technique (see Table 2).

Depending on the ablation technique applied, the cellular molecular mechanisms differ, but the overall mechanisms are similar.

The molecular mechanisms of the central region are classified into two categories: direct and indirect.

Thermal ablation-induced changes in cell membrane integrity are the central, direct cause of hyperthermia-induced cell death, including alterations in cell membrane fluidity and permeability, which in turn cause dysfunction of the intracellular microfilament/microtubule system and further lead to abnormalities in facilitated diffusion and active transport. Metabolite deposition and abnormalities in intracellular fluid homeostasis ultimately cause cell lysis.^68^ In addition, mitochondrial dysfunction is closely related to hyperthermia-induced cellular damage. Other possible mechanisms of intracellular damage due to hyperthermia include the disruption of RNA synthesis, lysosomal enzyme leakage, and Golgi damage.^68^^,^^69^

In addition to these direct causes, indirect or delayed cellular damage, including apoptosis, vascular injury, ischemic or ischaemia-reperfusion damage, and the release of lysosomal activators and cytokines, can occur after ablative therapy. These effects promote antitumor immunity and help with the overall effect of local treatment.

In the migrating region, a predominantly inflammatory cell infiltrate occurs, and these cells include neutrophils, macrophages, dendritic cells, NK cells, and specific T and B cells,^56^^,^^57^^,^^60^ which are present in distant tissues and the circulation while comprising the postablation microenvironment. The possible main reasons for this infiltration are as follows.

(1) Damage-associated molecular patterns (DAMPs) caused by programmed cell death RNA, DNA, and heat shock proteins (HSPs), especially HSP70 and high-mobility group protein B1 (HMGB1),^55^^,^^70^ cause intrinsic immunity that triggers acquired immunity.
(2) The extracellular matrix surrounding the tumor contains hyaluronic acid, collagen, and endothelial cells and releases proinflammatory cytokines,^55^ which in turn activate the inflammatory cytokines interleukin-6 (IL-6), tumor necrosis factor alpha (TNF-α), IL-1β, and IL-8 and vascular adhesion molecules.

### Ablative techniques activate specific immune systems in hybrid surgery

Radiofrequency ablation, microwave ablation, and cryoablation have different mechanisms of immune response, which may have different synergistic effects with surgery.

After radiofrequency ablation, the increase in HSP70 specificity plays a key role in stimulating the antitumor immune response. In animal experiments, HSPs derived from tumor cells or virus-infected cells can induce antigen-specific immunity.^71^^,^^72^ There is also evidence from clinical studies that HSPs are significantly elevated in the serum of patients after radiofrequency ablation, and the degree of elevation of serum HSP70 correlates with increased survival.^58^

Nevertheless, attention has also been given to the decreased number of CD4^+^ CD25^+^ FOXP3^+^ regulatory T cells in the post-ablative tumor microenvironment after radiofrequency ablation,^53^ which implies that peripheral tissues have decreased immune tolerance to tumor antigens and that this decreased tolerance results in stronger antitumor cellular and humoral immunity. Some clinical studies have shown that the number of tumor-specific T cells in peripheral tissues is increased in patients after RFA, and there is a correlation between these cell counts and the time of tumor-free survival (TFS).^54^^,^^56^

In these patients, the immune response may play a role in controlling other residual GGNs, increasing overall survival after hybrid surgery.

Like RFA, which is a form of energy ablation, MWA disrupts the homeostasis of a tumor cell’s internal environment by causing intracellular dipolar molecules to rotate and thus generate thermal energy; however, MWA is more efficient, with a greater range of action (up to 2 cm), and produces faster results.

However, compared with RFA, MWA more weakly stimulates local inflammation and activates intrinsic and acquired antitumor immunity, producing lower levels of the cytokines IL-1, IL-6, and HSP70^61^^,^^62^ because the higher temperature may destroy tumor-specific antigens. Recent studies have shown that MWA activates antitumor immunity by remodeling the immune microenvironment of tumour-draining lymph nodes (TdLNs), with the initial 4 days after ablation representing a critical window for immune activation.^73^ Emerging evidence further indicates that combining MWA with Flt3L, a dendritic cell-mobilizing agent, significantly increases the efficacy of PD-1 blockade therapy, suggesting a potential translational direction in ablation immunotherapy research.^74^

Cryoablation technology is currently less commonly used in China, mainly because of its relatively high cost. The mechanism of direct cellular damage in the region of lethal hypothermia (−20 to −40°C) by cryoablation differs from that of thermal ablation, in which the extracellular fluid water freezes earlier than the intracellular fluid water does,^75^ and the local microvasculature of the tumor is severely damaged, causing ischemic damage in the target area.

In nonlethal hypothermia, the activation of cellular autophagy and a postablation immune response play significant roles.^76^ As thermal ablation techniques (RFA and MWA) destroy the tumor-specific antigens left behind through thermal denaturation, thus weakening the postablation immune response, the preservation of the immunogenicity of tumor-specific antigens by cryoablation results in increased plasma inflammatory cytokine levels^59^^,^^77^ and increased postablation antigen accumulation in dendritic cells (animal models).^78^ From this perspective, cryoablation might be the future of ablative techniques in hybrid surgery.

### Decision-making in hybrid surgery

**Figure 4:**
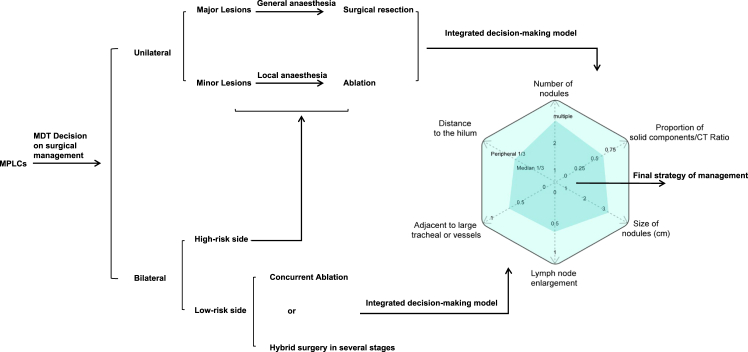
Decision-making model for hybrid surgery Represents our institutional protocol based on the MDT consensus and prior studies (Computed tomography-guided radiofrequency ablation combined with video-assisted thoracoscopic surgery for multiple pulmonary nodules: A retrospective study from the National Cancer Center in China). It is not yet validated prospectively but aligns with the NCCN guidelines for multifocal NSCLC.

The technique for decision-making in hybrid surgery is shown in Figure 4. For patients with MPLCs, a multidisciplinary team (MDT) makes the decision regarding surgical management. For patients with bilateral MPLCs, thoracic surgeons assess the risk associated with the lesions. We prefer the use of an integrated decision-making model to determine whether concurrent ablation or staged hybrid surgery should be applied to the low-risk side, whereas the high-risk side will follow the standard approach used for unilateral lesions. For unilateral lesions, major lesions are surgically resected, whereas minor lesions are treated with ablation. The type of lesion is determined by six key factors within the integrated decision-making model. The larger the area of the hexagon representing these factors is, the more likely surgical resection is to be favored. Conversely, smaller areas tend to favor ablation treatment. We must emphasize that synchronous hybrid surgery for bilateral lesions demands a rigorous multidisciplinary evaluation. Only low-risk contralateral nodules may safely undergo combined ablation and unilateral VATS. Inadequately assessed bilateral hybrid interventions risk precipitating bilateral pneumonectomy-like complications, despite isolated reports of such practices in earlier studies.^79^^,^^80^

### Surgical planning for hybrid surgery

VATS, as the treatment of choice for early-stage NSCLC, is still highly valuable in clinical practice. Therefore, we used surgery to treat the major lesions and combined surgery with thermal ablation to treat the minor lesions while retaining the advantages of anatomical surgical resection for pathological biopsy and lymph node dissection to achieve the “3M” goals of resolving as many lesions as possible at one time (“more lesions eliminated”), reserving as much lung function as possible (“more lung function reserved”), and preserving the possibility of receiving further treatment (“more chance for further treatment”).

**Figure 5:**
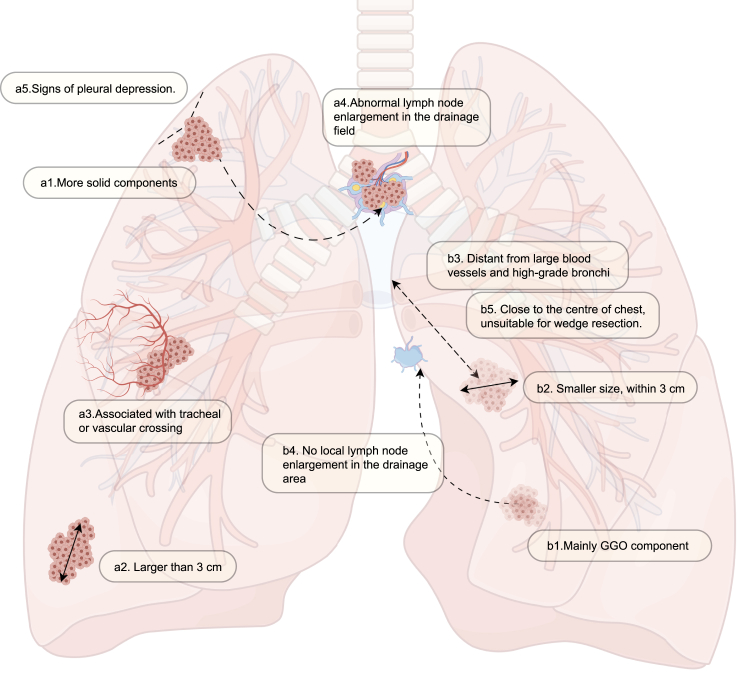
Surgical planning strategy for hybrid surgery

In clinical practice, we refer to the rules of thumb listed in the following text for the classification of major and minor lesions (Figure 5). The characteristics of the major lesions were as follows: (a1) multiple solid components; (a2) large size, usually larger than 3 cm^81^; (a3) association with tracheal and vascular crossing; (a4) abnormal lymph node enlargement in the drainage field; and (a5) signs of pleural depression.

The characteristics of the minor lesions are as follows (Figure 5): (b1) predominantly ground-glass density GGO (Ground-Glass Opacity) components; (b2) smaller size, usually within 3 cm; (b3) distance from large blood vessels and high-grade bronchi; (b4) no local lymph node enlargement in the drainage area; and (b5) a tumor location close to the center of the chest, which makes it unsuitable for wedge resection.

### VATS combined with ablation hybrid surgery: Technical route

The technical course of the combined procedure is as follows MDT decision-making on the operation, preoperative planning, preoperative anesthesia and disinfection, CT-guided puncture of the target lesion/electromagnetic navigation bronchoscopy (ENB)-guided approach to the target lesion, ablation, monitoring and adjustments during the ablation process, assessment of the postablation efficacy, change in position and resterilization, thoracoscopic puncture, resection of the planned site and assessment of the efficacy of the ablation, closure of the thoracic cavity, postoperative management, and CT follow-up of the efficacy of the procedure. The sequences of surgical resection and ablation are switchable; however, ablation before surgical resection is preferable because surgical treatment can address possible complications after surgical resection. Hybrid surgery can be performed with only general anesthesia or local anesthesia followed by general anesthesia.

#### Preoperative preparation

Patient indications were assessed by medical history, physical examination, and recent imaging outcomes. MDT evaluation was recommended, involving shared decision making (SDM) with the patient to formulate a final diagnosis and treatment opinion if necessary.^82^^,^^83^ The critical imaging evidence for developing a preoperative plan is a high-resolution contrast-enhanced CT of the chest that is completed within 1 month, which is important for defining the lung tumor size, morphology, internal structure, location, and adjacency. Lesions adjacent to major blood vessels (with a potential heat-sink effect) or critical nerves carry risks of incomplete ablation or adjacent tissue injury. A multidisciplinary evaluation by a team of ablation specialists is strongly recommended to assess procedural feasibility and safety. Notably, the following contraindications for hybrid surgery are areas of concern^84^:

Absolute contraindications—(1) platelet count <50 × 10^9^/L and (2) serious bleeding tendencies and coagulation disorders that cannot be improved in the short term (prothrombin time >18 s, prothrombin time activity <40%).

Relative contraindications—(1) severe cachexia and cardiopulmonary insufficiency; (2) significant infectious lesions on puncture routes; (3) severe COPD, emphysema, or pulmonary fibrosis; (4) severe pulmonary arterial hypertension; (5) patients using mechanical ventilation (ventilators) or an implanted cardiac pacemaker; and (6) patients with a psychotic episode.

#### Preoperative anesthesia and sterilization

Complex local anesthesia, including ropivacaine and lidocaine, with the use of a mask airway, is recommended according to the patient’s condition for CT-guided percutaneous ablation.^84^ As for ENB-guided endobronchial ablation, tracheal intubation and intravenous anesthesia are recommended.^79^^,^^80^^,^^85^ In principle, both general anesthesia and local anesthesia are applicable for ablation procedures, but each has distinct advantages and disadvantages. General anesthesia offers better patient comfort; however, it carries a higher risk of pneumothorax and may result in impaired lung expansion during ablation. Local anesthesia allows for patient cooperation, a shorter procedure time, and avoids intubation, but is associated with significant patient discomfort.

#### Minor lesion ablation

##### Ablation plan:

the ablation plan before surgery contains four elements. (1) use of the gross tumor region (GTR), which depends on the imaging-defined lesion area determined by preoperative high-resolution contrast-enhanced CT, to determine the location, size, morphology, and relationship with neighboring organs of the lesion.^86^ For ENB-guided ablation, computed tomography three-dimensional reconstruction is needed. (2) Confirmation of the puncture point and body surface positioning (using a sheet of paper with a metal grid while CT scanning). For ENB-guided ablation, confirmation of the tracheal route is needed. (3) Determination of the puncture path, i.e., the puncture path and its distance from the puncture point to the lesion. (4) Determination of the power and time for ablation according to the size of the lesion and the portion of the solid component, and use the preset parameters if there are no special circumstances.^81^ For ENB-guided ablation, computed tomography three-dimensional reconstruction is needed to plan the entry path of the ablation antenna.

##### Puncture target area (GTR)

after the patient is anesthetized and disinfected, according to the GTR of the presurgical ablation plan, from the puncture point on the body surface, the predetermined depth of puncture is gradually reached according to the puncture pathway, followed by the use of intrasurgical CT to observe whether the ablation needle reaches the GTR of the predetermined target area. For ENB-guided ablation, the ablation antenna reaches the target area through the guidance of ENB.

##### Ablation of GTR tissue

different ablation modes are used according to the size and number of tumors, such as (1) single GTR in one ablation session; (2) multiple GTR in one ablation session; and (3) multiple ablation needles/antennas and multiple GTRs in one ablation session. Specific ablation parameters are used according to the device type.

##### Monitoring and adjustment during the ablation process

CT is mainly used to monitor the “halo” sign after ablation, and when the boundary of the ablated GGO is 5–10 mm larger than that of the original GTR, the ablation is completed, and the needle/antenna is withdrawn.^86^ During the ablation process, it is also necessary to monitor whether the ablation needle is off-target, whether to adjust the power of the ablation needle, and whether there are intraoperative complications, such as bleeding, pneumothorax, etc., and to immediately switch to television-guided thoracoscopic surgery (VATS) if necessary. During the ablation process, the heart rate, blood pressure, and level of blood oxygen should be routinely monitored, including the patient’s respiration and bleeding, and symptomatic treatment should be given if necessary.

#### Position change and re-sterilization

The surgical team transfers the patient to the operating table. All procedures were performed under general anesthesia with double-lumen endotracheal intubation for selective lung ventilation.^87^ According to the patient’s intended surgical site, the patient can assume the lateral position, and according to the scope of sterilization for thoracoscopic surgery: anteriorly and posteriorly up to the midline, up to the clavicle and 1/3 of the upper arm, and down past the rib margins, sterilization with 0.5% PVP iodine should be performed, followed by the laying of a surgical towel (in the lateral position).

#### VATS major lesion resection

Anatomical lobectomy, sublobar resection, or wedge resection under thoracoscopic video display. First, the surgeon can confirm whether the minor lesions are completely ablated and whether there are complications such as bleeding or pneumothorax. The major lesion is subsequently resected via the following procedure. First, the anatomical structure around the lesion is clarified, the pulmonary vessels are bluntly separated, and the pulmonary vessels are ligated via an endoscopic knotter or cut with an endoscopic vascular suture cutter. The peribronchial lymph nodes are subsequently cleared. Surgeons should electrocoagulate small arterioles for hemostasis, using an endoscopic titanium staple to clamp the bronchial artery. After the bronchus is completely free, surgeons will select the surface to be severed, insert the endoscopic bronchial stump closure device, and cut off the bronchus. The pathology specimen of the main lesion is then placed into a specimen bag and removed through a small incision. Electrocoagulation or argon gas hemostasis is performed, the thoracic cavity is flushed, ventilation of both lungs is applied, the lungs are inflated to check whether there is any air leakage from the bronchial stumps, and the operation is concluded by placement of a drainage tube.

## Summarized perspectives on VATS combined with ablation

Some institutions have implemented the strategy of primary and minor lesion classification in the treatment of synchronous multiple primary lung cancer (sMPLC), and VATS combined with ablation surgery has been effective in the treatment of GGO-like lesions with a predominantly ground-glass component or lesions that do not have lymph nodes but have a certain risk of malignancy, as well as some nonnodular lesions, across numerous different pathologies or pathological stages. VATS ablation has been shown to be effective for several different pathological types or stages of GGO-like lesions with predominantly ground-glass opacities or for lesions without lymph node enlargement but with a certain risk of malignancy; VATS ablation is also effective for some nonnodular lesions, mainly in terms of more comprehensive management of GGO-like lesions and greater preservation of lung function after the procedure. Table 3 lists references concerning ablation combined with VATS hybrid surgery.

**Table 3:** Studies describing ablation combined with VATS hybrid surgery

Authors	Number of cases	Number of nodules/tumors	Number of nodules removed by VATS	Number of ablated nodules	Number and proportion of complications	References
Qu R et al.	11	37	16	21	1 (pneumothorax)	Qu et al.^80^
Bao F et al.	10	23	13	10	4 (air leakage∗ 1, haemoptysis∗ 2, lung infection∗ 1)	Bao et al.^88^
Zeng C et al.	57	216	120	96	10 (pneumothorax∗ 2, subcutaneous emphysema∗ 2, persistent cough∗ 6)	Zeng et al.^85^
Jiang N et al.	1 (single case report)	4	3	1	0	Jiang et al.^79^
Cui X et al.	91	More than 182	91	More than 91	49 (pneumothorax∗ 31 requiring close drainage∗ 7 (7.69%), pleural effusion∗ 6, hydropneumothorax∗ 5)	Cui et al.^89^
Liu B et al.	48	87	67	20	3 (air leak∗ 1, chylothorax∗ 1, massive pleural effusion∗ 1)	Liu et al.^90^

Regarding research on VATS combined with ablation hybrid surgery for lung cancers (Table 4), the main open-access ongoing clinical studies are registered by Chuan Huang et al. (Beijing Hospital), Jiayuan Sun et al. (Shanghai Chest Hospital), and Hecheng Li et al. (Ruijin Hospital) and are aimed to investigate the feasibility, perioperative safety, postoperative quality of life, and oncological efficacy of VATS combined with ablation for simultaneous multiple primary lung cancers. In addition to specific studies of VATS combined with ablation hybrid surgery for lung tumors, Jason A. (Beth Israel Deaconess Medical Center), Philippe L. Pereira, Hermann Aeber (University Hospital Tuebingen), Damian E. Dupuy (Meditronics), and Jennifer M. Kennedy (University Hospital Tuebingen) have conducted studies on the feasibility, perioperative safety, postoperative quality of life, and oncological efficacy of hybrid surgery for simultaneous or multiple primary lung cancers. These clinical studies are also informative for determining the procedural safety and prognostic benefits of combining VATS with lung ablation techniques for the treatment of lung tumors.

**Table 4:** Clinical trials on VATS combined with ablation hybrid surgery

PI	research period	Number of samples/cases	Main observational indicators	Subobservable indicators	Unique inclusion and exclusion criteria for projects	Shared inclusion criteria	Shared exclusion criteria	Reporting unit	Research numbers
Chuan Huang	2022.6.1–2024.12.31	50	Surgical feasibility, perioperative safety (complications), postoperative quality of life, oncological outcomes	–	∗∗Inclusion Criteria: Age ≥65 years old;	1. Age ≥18 years old. 2. Clinical diagnosis of synchronous MPLC according to the Martini-Melamed criteria; 3. patient can tolerate hybrid surgery according to MDT assessment 4. Lung tumor is resectable 5. Subjects voluntarily participate in the study and sign the written informed consent form at.	1. bronchoscopy cannot reach the minor lesion 2. Large blood vessels are within 2 mm or less from the contralateral minor lesion. 3. Patient cannot tolerate bronchoscopy 4. Any comorbidity that the investigator feels would interfere with the safety of the subject or the evaluation of study objectives 5. Pregnant or breast feeding. 6. Pacemaker, implantable cardioverter, or another electronic implantable device	Beijing Hospital	ChiCTR2300069053
Jiayuan Sun	2020.7–2025.12	30	Objective response rate (ORR) based on mRECIST criteria, assessed up to 1 year	PFS,OS,postoperative complication	∗∗Inclusion Criteria: (1) Ablation Lesion A is accessible/adjacent to bronchi and the size is ≤3 cm according to HRCT			Shanghai Chest Hospital	NCT04730453
Hecheng Li	2023.2–2025.12	172(86 + 86)	Perioperative complication rate	ORR,Perioperative surgery-related indicators, lung function, quality of life EORTC QLQ-LC29,economic cost,DFS	∗∗Inclusion Criteria: (1) Age ≥18 years and ≤80 years; (2) Bilateral simultaneous MPLC diagnosed by HRCT; (3) At least one minor lesion (6 mm ≤ diameter ≤20 mm, CTR <0.5) is located on the contralateral side of the major lesion and requires treatment after MDT discussion. (4) The ipsilateral minor lesion can be concurrently subjected to sublung lobectomy (5) ECOG PS score of 0–1 ∗∗Exclusion criteria: (1) Large blood vessels are present within 2 mm from the contralateral minor lesion.			Ruijin Hospital	NCT05662553
Walter J. Scott (Criteria not publicly available)	2002.6–2004.2	20	–	–	∗∗Inclusion Criteria: (1) Diagnosis of malignant non-small cell lung cancer (NSCLC). (2) Intraoperative needle biopsy confirmation allowed.(3)All tissue to be treated by radiofrequency tumor ablation must be completely contained within boundaries of planned lung resection			Fox Chase Cancer Center	NCT00039507
Jason A Beattie (Surgical pathology is only used to assess the effectiveness of ablation)	2022.6.1–2025.6.30	10	(1) Rate of planned ablations(Day 1) (2) Pathological changes in the tumor tissue: The % necrosis, % viable tumor, and % stroma/inflammation present in the targeted nodules after ablation(4 weeks).	(1) Histological changes in lung tissue outside the zone of predicted ablation: The % necrosis and of % stroma/inflammation will be described. (2) Immune-histochemical changes in the tumor tissue: Immune-histochemical evaluation including TTF-1, Napsin-A, p40, or other immune-histochemical assessments will be performed if needed on a per case basis.	∗∗Inclusion Criteria: (1) Subject with Stage I - II primary lung cancer (Solitary nodules up to 3 cm) as defined by previous pathology or ROSE. (2) Pathological proof of target nodule/tumor type and malignancy. (3) Target nodule/tumor which can be accessed via bronchoscopy and confirmed location with cone beam CT scan intraoperatively ∗∗Exclusion criteria: (1) Target nodule <1.0 cm. (2) Prior radiation or *neo* adjuvant chemotherapy of the target nodule/tumor. (3) Patients in other therapeutic lung cancer studies.			Beth Israel Deaconess Medical Center	NCT05281237
Philippe L Pereira, Hermann Aebert (Surgical pathology is only used to assess the effectiveness of ablation)	2004.4–2006.5	9	Rate of incompletely treated tumors	Pathologic tissue changes, rate of major and minor complications	∗∗Inclusion Criteria: (1) Maximum of 3 lung tumors. (2) Maximum tumor size less than 5 cm. (3) Must be able to receive standard surgery ∗∗Exclusion Criteria: (1) Pathological coagulation tests positive. (2) Maximum tumor size more than 5 cm. (3) Bilateral minor lung cancer with more than 3 tumors.			University Hospital Tuebingen	NCT00610844

## Prospects

### Prospects for the application of hybrid surgery

Prospects for the use of hybrid surgery are currently focused on 3 main areas: the optimization of intraoperative ablation techniques, advances in intraoperative guidance techniques, and immune-combination therapy with hybrid procedures.

### Upgrading of ablation techniques

Lung tumor ablation technology has gradually improved and developed. According to the characteristics of the ablation medium, this technology has now gradually developed into radiofrequency ablation, microwave ablation, and cryoablation, which have not yet been widely applied. In addition, with the further development of medical-industrial interdisciplinary fields, high-intensity focused ultrasound (HIFU) has also shown potential value in lung tumor treatment *in vitro* and in animal experiments.^91^ Photodynamic therapy (PDT), an important branch of laser ablation, can sensitize special nanoparticles to chemotherapeutic drugs through a photochemical reaction, thus impairing glycolysis process in tumor cells and enhancing the killing effect on tumor tissues. However, PDT has been used less often in lung tumors, and its effect and prognosis need to be systematically studied.^92^^,^^93^^,^^94^ As emerging ablation methods, reversible electroporation (RE) and irreversible electroporation (IRE) are considered to have important clinical potential because of their nonthermal effects and their ability to increase cell permeability in combination with cytotoxic drugs. However, owing to their high cost, these modalities are mostly used for unresectable local lesions that thermal ablation cannot eliminate as well. The difficulty of conducting clinical cohort studies involving these methods remains high.^95^^,^^96^^,^^97^

### Guiding technique progress

In terms of navigation strategies for lung tumor ablation, intraoperative navigation via CT or magnetic resonance imaging (MRI) is widely used in clinical practice. EBUS (endobronchial ultrasonography) intraoperative navigation is usually employed for the ablation of more dangerous tumors adjacent to the hilum, heart, and great vessels. A few institutions have applied more advanced three-dimensional imaging combined with ENB for intraoperative navigation shows potential for technical refinement. With the development of fusion imaging technology, the combination of multiple imaging signals to build guidance paths may become a trend, and the integration of augmented reality (AR) and virtual reality (VR) devices in surgery warrants exploration. Robot-assisted surgeries, such as da Vinci (Intuitive Surgical, Inc.), can also be combined with RFA for hybrid surgery.

### Immunotherapy for hybrid surgery

In recent years, the PD-L1 antibody atezolizumab has been reported to have a significant inhibitory effect on residual nodules in the lung region after RFA in clinical practice. However, no significant effect of the PD-L1 antibody atezolizumab has been observed for other tumors in the non-RFA ablation region. The practical application of the MWA technique has been reported in combination with camrelizumab.^98^^,^^99^ With the development of chimeric antigen receptor (CAR) technology, CAR-T cell systemic therapy was initially accepted for nonsolid tumors. Further research on CAR-T cell therapy in lung cancer has revealed that the altered tumor microenvironment after microwave ablation significantly enhances the antitumor effect of AXL-specific (AXL, Anexelekto, a kind of Receptor Tyrosine Kinase) CAR-T cells,^100^ suggesting that thermal ablation technology may have a synergistic effect with chimeric antigen therapy.^101^

The advancement of nanomaterial technology has significantly propelled the development of ablation combined with immunotherapy. As exemplified by the work of Xu, M et al., a multifunctional nanoplatform (UCILA) was ingeniously designed to integrate temperature monitoring with photothermal therapy. This platform simultaneously enables the high-resolution visualization of the tumor vasculature and real-time tracking of the metabolic distribution of UCILA *in vivo*. In animal studies, the synergistic application of UCILA-mediated photothermal ablation and CAR-NK immunotherapy produced a statistically significant improvement in survival rates, highlighting its dual capability for precise thermal dose regulation and systemic immune activation.^102^ Additionally, Jiang, Y et al. produced a nanoplatform (APNA) that promoted T cell infiltration through a photothermal effect.^103^ In the context of non-conventional ablation modalities, recent studies have shown that the hypoxia-tailored drug-loaded nanoplatform ADMOFs, designed for the hypoxic microenvironment following high-intensity focused ultrasound (HIFU) ablation, not only overcomes hypoxia-associated resistance to DOX (Doxorubicin) but also achieves a tumor volume inhibition rate of 77%.^104^ Numerous advancements in nanomaterial technology may provide valuable insights into tumor ablation immunotherapy.

### Prospects of basic research on hybrid surgery

Currently, the main basic research in hybrid surgery focuses on thermal ablation technology, which is mainly divided into biomedical directions related to tumor necrosis, apoptosis, and tumor immunity and engineering directions related to the distribution of the ablation thermal field, temperature field simulation, ablation antenna design, and temperature measurement technology.

In recent years, in the biomedical field, research on tumor thermal ablation has been progressing rapidly, and the academic community has a deeper understanding of the molecular mechanism and therapeutic synergistic effect of tumor thermal ablation.

However, in the field of engineering, much room for the development of a theoretical system of lung tissue thermal ablation technology still exists. (1) The idea of a multisource conformal thermal field was proposed by Phasukkit et al.^105^ through a finite element analysis of three-source microwave antennae and was initially verified in isolated bovine liver by Hoffmann et al.^106^ However, at present, there is no relevant research on the translation of these theoretical viewpoints into clinical practice, and such applications have not yet appeared in relevant studies. (2) The development of water-circulating internal cooling antennas effectively solves the problem of high rod temperatures, enabling the implementation of high-power, long-term, high-energy-level ablation, and the morphology of the ablation zone tends to be more spherical. However, relevant evidence for its clinical application and cost-effectiveness analysis needs to be obtained. (3) Owing to the complexity of the structure of human body tissues and individual variability, there is still considerable uncertainty in the solution of the heat transfer process of complicated biological tissues. Some scholars have carried out a series of studies on the change in the heat transfer properties of human tissues at different temperatures and the effect of the vascular tree on heat transfer,^107^ which provide a preliminary viewpoint on related issues. However, there are still some discrepancies between the simulation and experimental results of temperature field simulations, which require systematic cooperation between clinical and device engineering. (4) In terms of thermometry, invasive thermometry for tumors is relatively dangerous and inaccurate; however, currently, clinical thermometry technology is not available. The development of the current technology available for clinical ablation poses some technical difficulties that must be overcome. Zhou Zhuhuang et al.^108^ summarized the shortcomings of traditional ultrasound thermometry technology, such as the need for motion compensation. Because of the problems encountered with traditional thermometry technology, Camilo Correa-Gallego et al.^109^ reported that ultrasound and elastography can provide an accurate gross estimation of ablation zone size; however, these methods cannot be used to predict the degree of cellular injury and may cause the ultimate size of the ablation area to be significantly underestimated. This issue limits the application of ultrasonic temperature measurement technology. Indeed, the application of newer iterations of thermometry in ablation devices and comparative studies on the level and quality of ablation treatment and patient prognosis require the full cooperation of clinicians and technical engineers.

## Summary

The widespread adoption of chest LDCT, accelerated by COVID-19 screening protocols, has improved early lung cancer detection but concurrently increased psychosocial burdens from overdiagnosed pulmonary nodules. While conventional VATS anatomical lobectomy effectively treats early-stage malignancies, its associated pulmonary functional loss limits utility for multifocal lesions, particularly MPLCs. Hybrid VATS-ablation surgery addresses this dilemma by combining resection of dominant GGNs with concurrent ablation of satellite high-risk lesions. Early institutional studies demonstrate preserved lung function and oncological efficacy, supported by emerging evidence of synergies with CAR-T therapy and medical-industrial integration opportunities. This integrated approach leverages minimally invasive precision to balance curative intent and organ preservation, offering a tailored approach for multifocal disease. Nevertheless, clinical adoption requires resolution of interdisciplinary coordination challenges, standardization of ablation protocols, and validation through multicenter trials with extended follow up. Further exploration of ablation-induced immune modulation and techno-economic analyses will solidify its role in precision thoracic oncology.

### Limitations of the study

Current evidence on VATS-ablation hybrid surgery has several limitations. (1) The number and range limits of hybrid surgery for managing pulmonary nodules need to be further explored in practical clinical applications. (2) The coordination between different specialized departments (interventional, thoracic surgery, etc.) needs to be improved, and technical collaboration is necessary. (3) Patients’ acceptance of the strategy of hybrid surgical treatment needs to be improved through medical education. (4) Currently, there is a lack of large-scale, multicentre prospective clinical studies confirming the efficacy of hybrid surgery. (5) There is a lack of long-term (10–20 years) clinical follow-up data for patients who have undergone hybrid surgery. (6) There are many manufacturers of ablation therapy equipment, differences in equipment standards and performance pose major challenges to the promotion of this technology, and there is a need to establish recognized treatment norms and standards. (7) Systematic basic research is relatively lacking, and research on the ablation field distribution and postablation immune mechanisms needs further development.

## Acknowledgments

The authors acknowledge Home for Researchers (www.home-for-researchers.com) for providing technical support in figure illustration during this study. This work was supported by the 10.13039/501100001809National Natural Science Foundation of China (82203154), the Capital’s Funds for Health Improvement and Research (2024-2-4027), 10.13039/501100012166National Key R&D Program of China (2022YFC2407404), the National High Level Hospital Clinical Research Funding (CFA202502013, CFA202503003), the Cooperation Fund of CHCAMS (CFA202502013, CFA202503003).

## Author contributions

R.X., study conception, study design, data analysis and interpretation, statistical analysis, manuscript preparation, and manuscript editing. G.Z., study design, data analysis and interpretation, statistical analysis, manuscript preparation, and manuscript editing. N.R., Y.C., S.W., and C.Y. data acquisition. Q.X., Fe.T., L.Z., and J.H., funding acquisition supervision, and manuscript review.

## Declaration of interests

The authors declare competing interests.
